# Hospital Festivals and Meetings

**Published:** 1894-06-16

**Authors:** 


### HOSPITAL FESTIVALS AND MEETINGS.

### CITY OF LONDON HOSPITAL FOR DISEASES OF THE CHEST.

The City of London Hospital for Diseases of the Chest held its annual festival dinner at the Holborn Restaurant on Tuesday evening last. This East London institution is seriously in need of funds, not only for carrying on the work of maintenance, but in connection with the special building fund for the reconstruction of the drainage and sanitary appliances, and for making the ventilation more effectual. Much of the latter work has been carried out with conspicuous success, but much remains to do, to complete which some £4,000 is needed; and until it is forthcoming, progress must be postponed. As a proof that the financial position of the hospital is in a serious condition, it may be pointed out that the annual expenditure is not less than £11,000, apart from the special expenses, which this year arose from the fire in January last, while, after making full allowance for the annual subscriptions and awards from Hospital Sunday and Saturday Funds, &c., there is a sum of about £7,000 to be provided to meet the requirements of the twelve months. During the past three years the expenditure has exceeded the income by nearly £8,000, and the year 1893 closed with the liability of an undischarged loan of £1,500, which has since been increased to £2,000, a state of affairs which undoubtedly, as the committee admit, points, unless it is promptly remedied, to a limitation of the work of the institution. Certainly the dwellers in the East-end recognise the value of the work of the hospital; although their means may not always correspond with their will to assist, they, at any rate, in the shape of Friendly Aid and other societies do their best, thus perhaps setting an example to those in a far better position to help. Mr. F. D. Mocatta presided at the dinner, and the large company, numbering some 170, included Lieut.-Colonel Montefiore, Major Horace G Bowen, Rev. J. C. Mace, Messrs. Julian Hill, S. O. Gray, N. L. Cohen, F. H. Janson, J. D. Farrell, W. H. Herbert, Horace Mocatta, Cosmo Bevan, W. Ellis Hill, J. Norbury, junior, W. Mallett, R. H. Alexander, Morton Latham, B. E. Mocatta, C. G. Montefiore, G. F. Stutchbury, Barrow Emanuel, W. Burnett, and T. J. Isaac, together with Drs. Heron, Thorowgood, Vincent Harris, Chaplin, Hadley, Ware, Mason, and Carter. The societies represented were the Bethnal Green, the North Bow, the Albert and Victoria, the Mutual Friendly Aid, the East End, the Excelsior Philanthropic, the Good Intent Aid, the Mile End New Town, and the St. James's Philanthropic. The Royal toasts having been duly honoured, and the "Army, Navy, and Reserve Forces" proposed by Mr. Cosmo Bevan, and acknowledged by Lieut.-Colonel Montefiore and Major Horace Bowen,

The Chairman, who was very badly heard, proposed "Prosperity to the Hospital," and in so doing traced its history onward from its inception in 1848, in Liverpool Street, as an out-patient department, and its removal thence in 1855 to Victoria Park. The disease which they specially worked to combat was often caused by the insanitary dwellings of many of the poorer classes, but with the care, nourishment, and fresh air they obtained at the institution much was done to save the lives of the patients and avoid suffering in future. The institution also admitted a large number of cases of heart disease. Although he was opposed to special hospitals as a rule, he recognised that it was necessary to have such institutions for consumption and epilepsy. They had 164 beds, but at times in the year felt the paucity of the number; still, he might mention that in the past year 1,292 in-patients had been admitted, and 16,976 out-patients treated. After discussing the necessary steps for improving the sanitary arrangements and the making of structural additions, the Chairman said that it was a question whether they might not set apart, in time to come, a room for patients who could fully pay the requisite charges, and another for patients who could only pay part of them. (Cheers.) Concluding a long speech, which contained numerous suggestions to the authorities on points of management, the speaker said the Victoria Park Hospital was very much in want of money, but he hoped that they should succeed in removing the debt from the institution, whose expenses were very carefully watched, so that all extravagance was avoided. He trusted the public would become more alive to the wants of this institution, and would respond to their needs. And he certainly wished to point out the debt they owed to the members of the clubs established in the neighbourhood, who subscribed more than £1,000 a year and took so warm an interest in the work. (Cheers.) The toast having been drunk with great heartiness, the Secretary announced subscriptions to the amount of £1,844, including £100 from the Chairman. The other toasts, which concluded a long list, were "The Chairman," proposed by Mr. S. O. Gray; "The President (the Duke of Connaught), the Vice-Presidents, the Treasurer, and Committee," proposed by Mr F. H. Janson, and acknowledged by the Treasurer, Mr. Julian Hill; "The Medical Staff," proposed by Mr. Morton Latham, and acknowledged by Dr. Thorowgood; and "The Stewards," proposed by Mr. W. Ellis Hill, and acknowledged by Mr. J. A. Dickinson. The working men's societies contributed liberally to the funds, the Albert and Victoria subscribing 200 guineas, and the total from these sources being £1,144.

### NORTH-EASTERN HOSPITAL FOR CHILDREN.

The annual meeting of the governors of this hospital was held on June 6th at Devonshire House, Bishopsgate Street. The Chairman (Mr. R. Y. Godlee), in moving the adoption of the report, called attention to the very unsatisfactory state of the finances of the institution. If there should be no increase in the available funds the committee would, he said, be under the necessity of curtailing their work, as the mortgage on the hospital already amounted to £4,500. It might be thought that the generous legacy of £10,000 bequeathed by the late Mr. John Horniman should have freed them from their difficulties, but, according to the wishes of the testator, the whole amount had been invested, and only the interest, therefore, was available for current expenses. In aid of the institution, several drawing-room meetings were being held, which it was hoped would materially assist in reducing their debt; and the committee trusted that their special appeal for funds would be responded to by the subscribers to the hospital. Other speakers were: Mr. J. B. Scott, who proposed the election and re-election of the members of the committee for the ensuing year; Mr. Arthur Wenham; and the Rev. W. G. Morcom.

The report, although not financially a cheerful one, shows an increase in the work done by the institution, the in-patients during the past year having numbered 694, and the attendances of out-patients 55,538.

### HOSPITAL FOR WOMEN, SOHO SQUARE.

The annual general meeting of the governors of this hospital was held on June 7th, with the Duke of Westminster in the chair. In moving the adoption of the report, the Chairman said that it was very gratifying to be able to record the completion of the new wing, the building of which had been necessitated by the increasing work of the hospital. The nurses would be more comfortable in their new quarters, and of all people in the world he thought nurses should be well cared for. They had to make the usual appeal for funds, for they could not always depend on legacies to eke out their income. Sir Rutherford Alcock, in appealing for funds, said that the management of the hospital had not been extravagant. They had waited to build until they had sufficient funds in hand, and were not now sending round the hat for work already done. They must hope in the future to get their income to equal their current expenditure. Other speakers were Mr. A. H. Smith, M.P., Colonel Boyle, Captain Welbe, and the Rev. J. H. Cardwell, and several ladies, including the Duchess of Westminster and the Countess of Romney, were present. In spite of the hospital having been closed for six weeks of the year for the purpose of reflooring, the number of in-patients—615—showed an increase of 35 over the previous year; and the attendances of out-patients were 29,983, or an increase of 1,000 attendances.

